# The Utilisation of Payment Models Across the HIV Continuum of Care: Systematic Review of Evidence

**DOI:** 10.1007/s10461-021-03329-2

**Published:** 2021-06-28

**Authors:** Tiago Rua, Daniela Brandão, Vanessa Nicolau, Ana Escoval

**Affiliations:** 1 King’s Health Economics, King’s College London, London, UK; 2 Escola Nacional de Saúde Pública, Nova University, Lisbon, Portugal

**Keywords:** Acquired Immunodeficiency Syndrome, HIV infections, Multimorbidity, Healthcare Financing, Payment Models, Economic, Síndrome de Inmunodeficiencia Adquirida, Infecciones por VIH, Multimorbilidad, Financiamiento Sanitario, Modelos de Pago, Económico

## Abstract

The increasing chronicity and multimorbidities associated with people living with HIV have posed important challenges to health systems across the world. In this context, payment models hold the potential to improve care across a spectrum of clinical conditions. This study aims to systematically review the evidence of HIV performance-based payments models. Literature searches were conducted in March 2020 using multiple databases and manual searches of relevant papers. Papers were limited to any study design that considers the real-world utilisation of performance-based payment models applied to the HIV domain. A total of 23 full-text papers were included. Due to the heterogeneity of study designs, the multiple types of interventions and its implementation across distinct areas of HIV care, direct comparisons between studies were deemed unsuitable. Most evidence focused on healthcare users (83%), seeking to directly affect patients' behaviour based on principles of behavioural economics. Despite the variability between interventions, the implementation of performance-based payment models led to either a neutral or positive impact throughout the HIV care continuum. Moreover, this improvement was likely to be cost-effective or, at least, did not compromise the healthcare system’s financial sustainability. However, more research is needed to assess the durability of incentives and its appropriate relative magnitude.

**Supplementary Information:**

The online version contains supplementary material available at 10.1007/s10461-021-03329-2.

## Resumen

La creciente cronicidad y multimorbilidades asociadas con las personas que viven con el VIH han planteado importantes desafíos para los sistemas de salud en todo el mundo. En este contexto, los modelos de pago tienen el potencial de mejorar la atención en un espectro de condiciones clínicas. Este estudio tiene como objetivo revisar sistemáticamente la evidencia de los modelos de pagos basados ​​en el desempeño aplicados al dominio del VIH. Las búsquedas bibliográficas se realizaron en marzo de 2020 utilizando múltiples bases de datos y búsquedas manuales de artículos relevantes. Los artículos se limitaron a cualquier diseño de estudio que considere la utilización en el mundo real de modelos de pago basados ​​en el desempeño aplicados al dominio del VIH. Se incluyeron un total de 23 artículos de texto completo. Debido a la heterogeneidad de los diseños de los estudios, los múltiples tipos de intervenciones y su implementación en distintas áreas de la atención del VIH, las comparaciones directas entre diferentes estudios se consideraron inadecuadas. La mayoría de la evidencia se centró en los usuarios de la salud (83%), buscando afectar directamente el comportamiento de los pacientes basándose en los principios de la economía del comportamiento. A pesar de la variabilidad entre las intervenciones, la implementación de modelos de pago basados ​​en el desempeño generó un impacto neutral o positivo en todo el proceso de atención del VIH. Además, es probable que esta mejora sea costo-efectiva o, al menos, no comprometa la sostenibilidad financiera del sistema de salud. Sin embargo, se necesita más investigación para evaluar la durabilidad de los incentivos y su magnitud relativa apropiada.

## Introduction

The HIV / AIDS epidemiological evolution remains challenging across the world. Whilst low to intermediate-income countries have focused on tackling new infections and the spread of the disease, the epidemiological evolution in high-income countries has led to people with HIV living longer, and with multiple comorbidities [1]. This increase in chronicity and multimorbidities associated with people living with HIV is posing important challenges to health systems across the world [2]. In fact, given the increasing medical costs per person living with HIV infection [3] and the ever-growing budget constraints, transformational changes in both the HIV care delivery and payment models are required [4].

Multiple payment models have been tested in healthcare, typically characterised based on its: (i) recipients; and (ii) payment unit(s). First, concerning the model’s recipients, payment models are typically utilised to reimburse healthcare providers for their services. However, payment models are also used to cascade down institutional incentives to the individual level, either to incentivise healthcare professionals or the healthcare users themselves. Second, regarding the unit payment, historical unit payments centered on retrospective cost reimbursements have been progressively replaced with prospective unit payments, particularly based on activity (e.g. fee-for-service, bundled payments per episode or per diagnosis) or capita. More recently, payment models have moved from volume to value-based unit payments, with the implementation of performance-based payment models which aim to improve quality of care and decrease overall costs [5]. Different unit payments present diverse advantages and disadvantages, leading to the coexistence of multiple unit payments across different production lines (e.g. ambulatory care, admissions), care providers or even healthcare systems [2, 3]. As an example, if the model’s aim is to improve healthcare access, then volume-based unit payments may be appropriate, whilst if the aim is to enhance disease prevention, the capita unit payment system may be more appropriate. In essence, the payment model’s structure should be aligned with its overall aim.

Given the high mean healthcare cost per person living with HIV infection [3], payment models must balance: (i) the financial risk for all stakeholders; (ii) the promotion of quality of care and care integration [1, 2]; and (iii) multiple recipients, from healthcare providers, healthcare professionals or even the people living with HIV themselves [6]. For these reasons, novel HIV payment models tend to be performance-based as opposed to volume-based.

Over the past decade, multiple reviews evaluated the impact of performance-based HIV payment models [7–15], which can be grouped according to: the healthcare setting considered (low to intermediate-income countries vs high-income countries); and the area of HIV care considered (from HIV testing and HIV prevention to adherence to treatment). Notably, a systematic literature review published in 2017 assessed the implementation of performance-based payment models in low-income countries [13]. Only four studies were included in this review and the authors concluded there was little evidence regarding the real-world implementation of HIV payment models.

The present manuscript builds upon previous reviews but presents four innovative features. First, contrary to previous studies that only consider high or low-income countries, this review did not exclude evidence based on the study’s country of origin. Second, this review aimed to capture any performance-based payment model regardless of its recipient (healthcare providers, health professionals or healthcare users). Third, the entire HIV continuum of care was considered, from prevention to the treatment of people living with HIV. Previous evidence tended to focus on a specific area, either HIV prevention, adherence to treatment or the delivery of antiretroviral treatment (ART). Fourth, linked to the increasing chronicity of HIV in high-income countries, the review included search terms concerning the use of payment models in the context of multimorbidity.

As a corollary, this systematic literature review aims to summarise the real-world evidence around the implementation of HIV performance-based payment models and provide a solid foundation on which to base decisions from healthcare policy makers across multiple healthcare settings.

## Methods

A systematic literature review was conducted to analyse any empirical evidence around the implementation of payment models in the field of HIV / AIDS. This systematic review was conducted in accordance with the Preferred Items for Systematic Reviews and Meta-Analyses (PRISMA) statement using a pre-defined protocol (International Prospective Register of Systematic Reviews, PROSPERO identification number CRD42020167941) [16].

### Search Strategy and Databases

The search strategy was defined based on the PICOS (Population, Intervention, Comparator, Outcome, Study design) framework, as summarised in Table 1. The following electronic databases were searched: MEDLINE (PubMed), SCOPUS, Emerald, Web of Science, and the Cochrane Library. The search strategy (Online Appendix 1) was consistently used in the databases searched, with only minor adjustments specific to the database searched. The use of truncation, wildcards, and Boolean logic aimed at maximising the number of relevant articles. In addition, references cited in the identified papers were also examined. In summary, the search strategy aimed to include studies of any design which evaluated the clinical and/or financial impact of implementing real-world payment models across the HIV continuum of care.

**Table 1:** PICOS strategy considered in the systematic literature review

Population	Adults (over 18 years old) with HIV/AIDS or adults considered eligible for HIV prevention or screening programmes
Intervention	Any type of performance-based payment model
Comparator	No additional payment model (e.g. existing standard of care)
Outcomes	Clinical outcomes, both process (e.g. proportion of HIV patients in antiretroviral therapy) and result outcomes (e.g. proportion of HIV patients with supressed viral load)
	Financial outcomes (e.g. mean cost per treated patient, mean cost per provider or patient included in the payment model)
Study design	Any study regardless of the methodology used (e.g. qualitative study, quantitative study, mixed methods study) published from 2008 onwards

### Inclusion and Exclusion Criteria

The inclusion and exclusion criteria were defined as per the PICOS strategy (Table 1). As inclusion criteria we considered: (i) studies including adults living with HIV/AIDS aged 18 years or older (studies including children were included if the data had been disaggregated by age group for adults aged ≥ 18 years); (ii) studies, regardless of its design, in which clinical and/or economic outcomes related to the implementation of performance-based payment models were considered; (iii) studies published from 01/01/2008 onwards; and (iv) studies published in English. The age group was limited to adults as performance-based payment models are typically aimed at adults who live with the HIV infection or are at-risk of contracting the infection. With regards to the time frame, the utilisation of behavioural economics applied to the HIV domain has been quite recent, particularly after the introduction of HIV antiretroviral drugs. As an exclusion criterion, non-human studies were excluded.

### Screening and Data Extraction

**Fig. 1:**
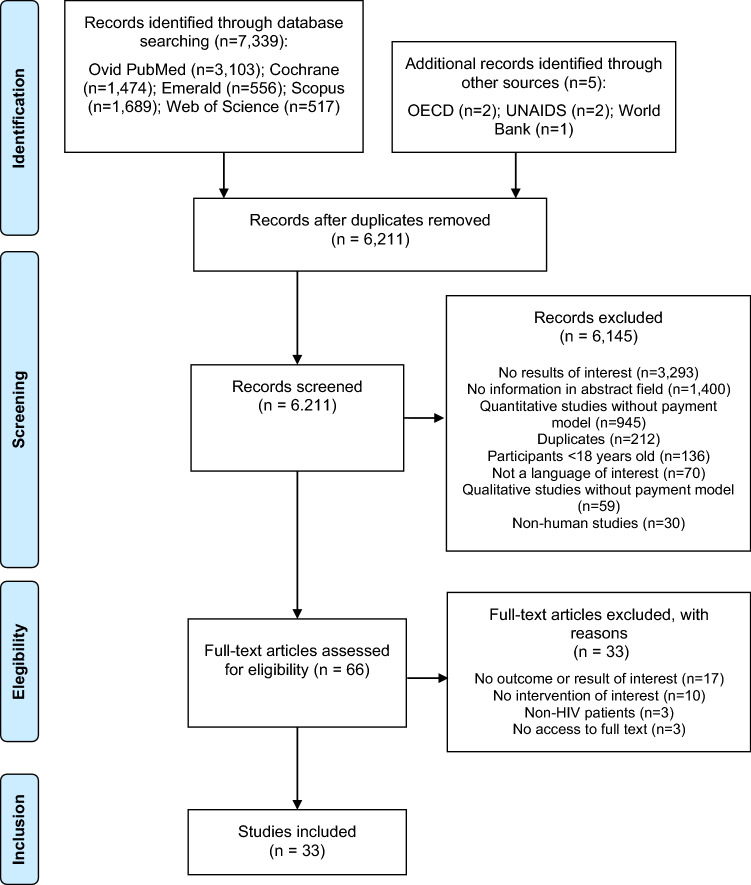
PRISMA flow chart summarising the selection process of relevant studies

Figure 1 illustrates the process of study selection and data extraction. Abstracts were independently screened by two reviewers (TR, DB) and included or excluded as per the screening protocol. Full texts of selected articles were retrieved and independently assessed by two reviewers (TR, DB) for consistency in alignment with the review’s inclusion and exclusion criteria. Both investigators indicated the reason(s) to include or not include each study in the systematic review. Any disagreements in the screening of abstracts and data extraction from full text papers were reached through consensus and, if not possible, by a third reviewer (VN). The data extraction methodology considered the collation of the following data in an electronic form spreadsheet (using Microsoft Excel):

- Study description: record number, authors, study title, year, journal, country, study period, source of funding (e.g. public, private, social sector), reference, study methods;
- Study and participant details: study objectives, study design, study inclusion and exclusion criteria, recruitment procedures used, participants’ allocation methods, population description (e.g. age, sex, socio-economic status of participants, sample size, CD4 count, ART status);
- Intervention and reported outcomes: intervention and comparator, primary and secondary outcomes measures, period of follow up, subgroup analysis, subgroup details (if applicable), sensitivity analysis;
- Key findings, summary of study strengths and limitations.

All bibliographic references were managed (e.g. elimination of duplicates) using the bibliography management software Zotero.

### Quality Assessment

Two reviewers (TR, DB) assessed the risk of bias of each included study using the ‘Mixed Method Appraisal Tool (MMAT)’ [17]. MMAT contains 19 core criteria in a quality scoring system which are grouped into five methodological categories, according to the study design: qualitative, quantitative with randomisation, quantitative without randomisation, quantitative descriptive studies and mixed methods.

## Results

A total of 23 full text studies were included in the review (see Fig. 1). These presented different methodologies in terms of: study design; country of origin; recipient of the intervention; type of intervention; clinical evidence; and economic evidence. For these reasons it was not deemed appropriate to summarise the evidence using meta-analyses and instead a descriptive synthesis of evidence was undertaken. Table 2 summarises the key methodological information extracted from the 23 studies included. Table 3 illustrates the clinical and economic results from the 23 studies included in this systematic review (for detailed information please refer to Online Appendix II). The evidence in the appendices are listed in chronological order (newest to oldest) and grouped per study design.

**Table 2:** Summary of the population, area of HIV and interventions considered per study included

Reference and country of origin	Population (number and inclusion criteria)	Area of HIV continuum	Intervention and comparator	Overall article quality
*Randomised controlled trial*				
Choko et al. (2019)[18]	Population: Male partners of pregnant women who had their first prenatal visit. 1: 1 ratio in all 5 groups were considered, obtaining a total sample of 2,349 individuals	Prevention: male circumcision rate	Intervention: 4 incentive strategies, 3 with financial incentives (assignment of rapid HIV tests with $ 3 and $ 10 conditional fixed incentives and a third incentive based on a $30 lottery) and a strategy with non-financial incentives (quick test + two calls follow-up phone calls)	High
Country of origin: Malawi	Inclusion: pregnant women who had their first prenatal care appointment	Link to treatment care	Comparator: clinical practice with no financial incentives	
El-Sadr et al. (2019)	Population and Inclusion: Participants in the HPTN 065 study (El-Sadr et al. 2017)	Care retention (continuity of care)	Intervention: utilisation of individual financial incentives around continuity of care (e.g. attending appointments) and viral suppression	High
Country of origin: USA		Viral load suppression	Comparator: clinical practice with no financial incentives	
Thirumurthy et al. (2019)	Population: 400 participants (203 in the intervention group and 197 in the control group)	Care retention	Intervention: received financial incentives conditional for viral suppression at 6, 12 and 24 weeks, with the value of the incentive to scale from $ 4 to $ 12.5	Intermediate
Country of origin: Uganda	Inclusion: aged 18 + , living in one of the four communities considered in the study, with a positive result for HIV, and who were starting or already receiving ART	Adherence to treatment and viral suppression	Comparator: viral load tests and counselling	
Kadota et al. (2018)	Population: 805 HIV positive individuals recruited at 3 clinics	Link to care and ART initiation	Intervention: two intervention groups:(1) food assistance; (2) money transfers	High
Country of origin: Tanzania	Inclusion: Adult HIV patients, with food insecurity, and on ART initiation ≤ 90 days before enrolment in the study	Care retention	Comparator: usual care	
Maughan-Brown et al. (2018)	Population: 84 individuals equally distributed between groups	Link to care and ART initiation	Intervention: usual care + voucher (exchangable for monetary value if ART started in the following 3 months)	Intermediate
Country of origin: South Africa	Inclusion: HIV-diagnosed adults referred for ART by a mobile health clinic in Cape Town who had never previously had ART		Comparator: usual care (telephone follow-up advice from staff to encourage care promotion)	
Mills et al. (2018)	Population: Participants from two rural districts of Uganda, recruited through AIDS centres and home visits to patients who had not visited a centre recently	Prevention	Intervention: unconditional monetary grants, with or without financial advice: Group T1: unstructured grant; Group T2: mental planning + grant	Intermediate
Country of origin: Uganda	Inclusion: HIV positive, aged between 18 and 60 years old, living in the two rural districts considered	Link to care and ART initiation		
		Viral suppression	Comparator: Group T3: control (no incentives); and Group T4: expectations / control (participants were told they would receive a monetary grant 12 months after the program started—after the follow-up interview)	
Montoy et al. (2018)	Population: 8,715 patients in an urban emergency department (San Francisco)	Testing and screening	Intervention: $ 1 incentives, $ 5 incentives, or $ 10 incentives (each of these groups, with 3 subgroups—opt-in, active choice, opt out test)	High
Country of origin: USA	Inclusion: aged between 13–64 years; being able to give consent for HIV testing and study inclusion in the study; speak English or Spanish		Comparator: without incentives (also with 3 subgroups—opt-in, active choice, opt out test)	
Chamie et al. (2018)	Population: A total of 2,527 participants had complete data and were considered for analysis	Testing and screening	Intervention: (1) incentives included in the loss: participants who had won a prize were later told they would lose that prize if they did not take the test; 2) lottery-based incentives: participants who had an HIV testing automatically enter a lottery and had the opportunity to instantly win prizes	High
Country of origin: Uganda	Inclusion: adult men (≥ 18 years old) referred to in the censuses, who lived in the community for ≥ 6 months in the year prior to referral and who had no intention of leaving the community in the following 3 months			
Alsan et al. (2017)	Population: 110 HIV-infected adults with a plasma viral load (pVL) > 200 copies / mL despite ART (n = 21 adults in the professional visit incentive group; n = 19 adults in the choice incentive group, n = 70 in the passive control group)	Care retention and adherence to treatment	Intervention: (1) incentive group to visit professionals (US $30 incentive after participating in each scheduled visit to professionals); (2) incentive choice group (commitment contract that makes the payment of US $ 30 conditioned both to participation in the scheduled visits of professionals and adherence to ART)	Intermediate
Country of origin: USA	Inclusion: the HIV-1 plasma RNA viral load over > 200 copies / mL measured in the previous 18 months and at least 6 months after starting the current ART regimen	Viral suppression	Comparator: routine care without any incentive	
El-Sadr et al. (2017)	Population: 1,159 HIV patients (new HIV patients or unknown to healthcare in the past 12 months) were recruited to assess adherence to healthcare (389 in New York and 770 in Washington) and a total of 16,208 patients with HIV (established HIV patients) were recruited to assess viral load suppresion (9,703 in New York and 6,505 in Washington)	Care retention and adherence to treatment	Intervention: healthcare providers were randomised to provide financial incentives to HIV patients. Participants received: (1) a $ 125 voucher ($ 25 for conducting CD4 tests and $ 100 for an HIV planning medical consultation); and / or (2) $ 70 voucher for HIV testing with suppressed viral load (CD4 < 400 copies / mL), with a maximum of one voucher each quarter	High
Country of origin: USA	Inclusion: Health providers, not their users, were randomised to the intervention and control group	Viral suppression	Comparator: healthcare providers randomised not to give financial incentives (i.e. follow usual care)	
Stitzer et al. (2017)	Population: post hoc secondary analysis of the HOPE study (Metsch et al., 2016)	Care retention	Intervention: please refer to Metsch et al. (2016) – see below	Intermediate
Country of origin: USA	Inclusion: please refer to Metsch et al. (2016) – see below		Comparator: please refer to Metsch et al. (2016) – see below	
Metsch et al. (2016)	Population: 801 patients with HIV and substance abuse from 11 hospitals	Viral suppression	Intervention: 2 groups: (1) received a structured patient navigation intervention (up to 11 care coordination sessions, with case management and motivational interview techniques over 6 months) (n = 266); (2) structured patient navigation intervention and financial incentives (up to US $ 1,160) (n = 271)	High
Country of origin: USA	Inclusion: several criteria are mentioned (e.g. inpatients with HIV infection, being 18 years or older)		Comparator: usual care (n = 264)	
de Walque et al. (2015)	Population: HIV patients from different clinics randomised to the intervention and control group	Testing and prevention	Intervention: randomised health care providers (n = 10) received incentives in the field of HIV prevention and treatment (organisational incentive)	Intermediate
Country of origin: Rwanda	Inclusion: Health care providers that treat patients with HIV infection were randomised		Comparator: randomised health care providers to receive an increase in fixed financing equivalent in magnitude to the intervention model (n = 14)	
de Walque et al. (2012)	Population: 2,399 participants between the ages of 18 and 30 from certain ten geographical areas in Tanzania	Prevention	Intervention: two groups, with varying magnitude of incentive: (1) group with low level of incentive ($ 30 total incentive) and (2) group with high level of incentive ($ 60 total incentive). In both cases, the incentive was conditional on a negative test result every 4 months	Intermediate
Country of origin: Tanzania			Comparator: usual care with no incentives	
Barnett et al. (2009)	Population: 86 individuals, with 66 of these with less than 80% adherence to their ART (34 were allocated to the incentive group with a voucher and 32 were allocated to the comparison group)	Care retention and adherence to treatment	Intervention: incentives with vouchers conditional on adherence to ART. The participant could accumulate earnings before choosing a redeemable voucher for purchases, meals or other	Intermediate
Country of origin: USA	Inclusion: HIV positive patients on methadone maintenance were eligible if they had been taking ART for at least 1 month		Comparator: weekly lottery was used to reduce dropouts among these participants, (3 in 1 chance of winning a small prize and a 350 in 1 chance of winning $80)	
*Non-randomised prospective study*				
Belenky et al. (2018)	Population: a total of 801 women with HIV and different insurance coverage	ART initiation	Intervention: change to the HIV drug financing model: HIV patients with Medicare / Medicaid double coverage, eligible for the transition to the Medicare Part D system in Jan 2006	High
Country of origin: USA	Inclusion: women with HIV with at least one HIV follow-up visit; and included in the Medicare / Medicaid program (double coverage) or only in the Medicaid program with adherence to the Medicare Part D program in January 2006	Viral suppresion	Comparator: HIV patients and Medicaid coverage and no additional insurance in 2005	
Brantley et al. (2018)	Population: HIV patients recruited from 3 clinics in the State of Louisiana in the USA (n = 2076)	Adherence to ART	Intervention: assigning multiple individual card incentives: an initial incentive of $ 50, $ 20 for each medical consultation, $ 10 for each laboratory exam, $ 10 for referral to the service and $ 75 for keeping the viral load below the target (n = 2076)	Intermediate
Country of origin: USA	Inclusion: HIV patients with at least one laboratory test in the year prior to recruitment	Viral suppresion	Comparator: HIV patients who did not integrate the individual incentive model	
Rajkotia et al. (2017)	Population: 134 health institutions	Prevention	Intervention: organisational incentive model based on performance-based unit payments	High
Country of origin: Mozambique	Inclusion: health institutions in the provinces of Nampula (north) and Gaza (south), certified by the Government of Mozambique to provide ART and prevention of mother-to-child transmission of HIV		Comparator: only standard input-based financing (without performance-based incentives)	
Foster et al. (2014)	Population: A total of 11 adults with an average age of 19 years and 8 months	Viral load suppresion	Intervention: allocation of individual financial incentives in combination with consultation with a clinical psychologist and / or specialist nurse trained in motivational interviewing techniques (up to a total incentive of £ 200 conditional on sustained reductions in viral load and participation in motivational consultation)	Low
Country of origin: UK	Inclusion: patients with vertically acquired HIV who transitioned from paediatric services to adults with: CD4 count < 200 cells / µL); not taking ART despite several attempts to start treatment; and with the intention of resuming ART		Comparator: no comparator / control group	
*Modelling studies (cost-effectiveness studies)*				
Wagner et al. (2020)	Population: Patients in the emergency department of the Zuckerberg San Francisco General Hospital and Trauma Centre (Montoy et al., 2015)	Prevention	Intervention: small immediate financial incentives, opt-in / opt-out testing and a combination of both schemes	Intermediate
Country of origin: USA	Inclusion: age between 13–64 years; being able to give consent for HIV testing and the study; speak English or Spanish. Patients with previous HIV diagnosis were excluded	Testing and screening	Comparator: standard option (offering tests through an inclusion or exclusion regime)	
Adamson et al. (2019)	Population: HIV patients in the Bronx (New York) and Washington DC (n = 16.208)	Viral load suppression	Intervention: $ 70 vouchers for patients with CD4 viral load < 400 copies / mL	High
Country of origin: USA	Inclusion: HIV patients who received incentives based on a randomised clinical trial (El-Sadr et al. 2017)		Comparator: no comparator / control group was considered	
Stevens et al. (2018)	Population: participants in the Link4Health randomised clinical trial	Prevention	Intervention: (1) Point-of-care CD4 + count testing; (2) Accelerated ART initiation; (3) Mobile phone appointment reminders; (4) Care and prevention package including commodities and informational materials; and (5) Non-cash financial incentive	High
Country of origin: Swaziland		Testing and screening	Comparator: usual care with no incentive	
Heymer et al. (2012)	Population: Based on HIV epidemiology in South Australia (data from 2004 to 2008)	Prevention	Intervention: removal of negative financial incentive (co-payment)	High
Country of origin: Australia	Inclusion: not mentioned		Comparator: usual care with co-payments

**Table 3:**
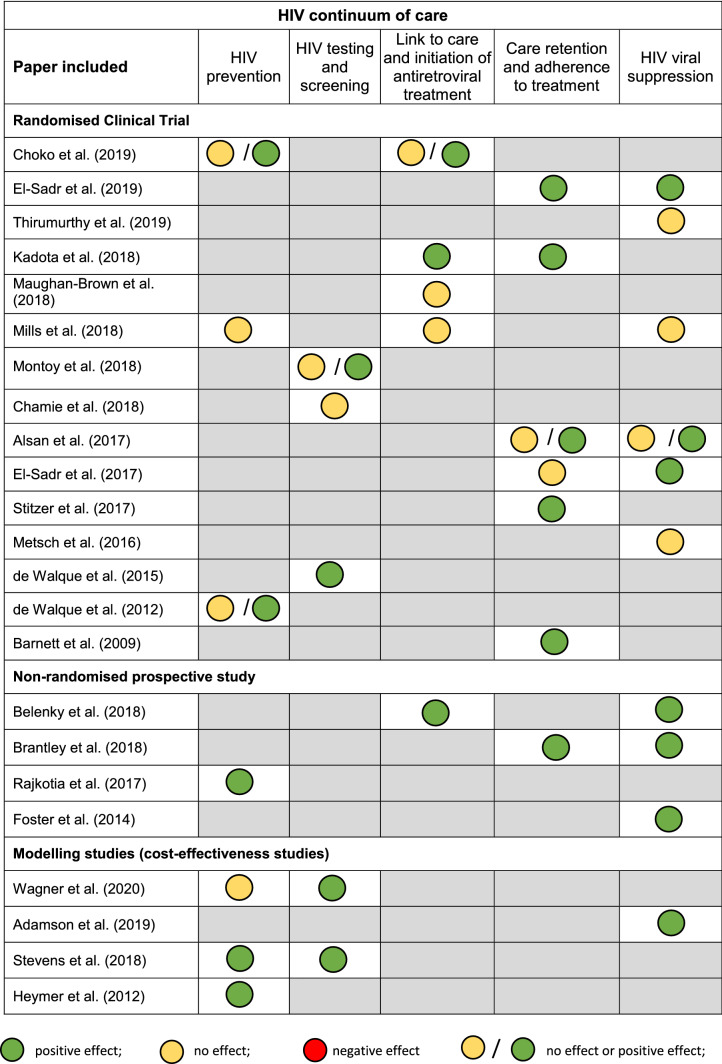
Impact of the different payment models across five areas of HIV care

### Study Design

Of the 23 articles included, 15 (65%) articles reported randomised clinical trials [19–33], 4 (17%) non-randomised empirical studies [34–37] and 4 (17%) quantitative descriptive studies with cost-effectiveness analyses [38–41].

### Country of Origin

The studies included were classified according to their country of origin (see Table 2), given that interventions with payment models have the potential to be diversified according to the country in which the intervention is implemented. Thirteen out of 23 (57%) papers were derived from countries with high-income levels, specifically the USA (11 studies), the UK and Australia (1 study each). The remaining 10 (43%) articles were derived from several African countries with low to medium-income levels: Uganda (3 studies), Tanzania (2 studies) and Malawi, South Africa, Mozambique, Swaziland and Rwanda (1 study each).

### Intervention’s Recipient

The payment model may also vary depending on its recipients: (i) health organisations; (ii) health professionals; and (iii) health care users themselves. A total of 19 of 23 articles (83%) focused on health users, followed by 7 of 23 (30%) articles with an emphasis on health organisations and no evidence on healthcare professionals. Three articles simultaneously incentivised health organisations and health care users.

### Type of Intervention

Different studies have considered various interventions, particularly regarding the model’s unit payment. However, the evidence presented a common denominator as all interventions that aimed to change the behaviour of health users deployed positive financial incentives.

All seven papers that focused on health organisations considered the use of incentives based on pay-for-performance. However, the rationale inherent as to how incentives aimed to change organisational behaviours varied, as did the area of ​​HIV on which the incentives were focused. At the health care users’ level, the empirical evidence (19 articles) concentrated mostly on the use of financial incentives to change behaviours across the various areas of HIV. These financial incentives were always positive incentives, with a view of promoting appropriate behaviour, and not negative incentives aimed at inhibiting incorrect behaviours. However, these incentives varied greatly in terms of structure, with financial incentives being attributed as: (i) a fixed component, where incentives were paid to all participants regardless of the results obtained; (ii) based on a lottery, where only one or more participants received incentives based on a random draw; and / or (iii) a conditional component, where there is only room for payment (partial or total) depending on the achievement of certain objectives, usually around clinical indicators associated with HIV management. It should be noted that most studies in low-income countries considered the use of fixed incentives whilst in high-income countries these tended to be conditional. Moreover, different incentive structures were not mutually exclusive, as some payment models were hybrid, with fixed incentives complemented by conditional ones. In addition, in countries with low to intermediate income levels, some models included the use of non-financial incentives, such as the allocation of food items or food vouchers.

### Clinical Evidence

The clinical evidence presented in the systematic review was grouped along the continuum of HIV care according to five areas: (i) HIV prevention; (ii) HIV testing and screening; (iii) link to HIV care and initiation of antiretroviral therapy; (iv) retention in health care and adherence to treatment; and (v) viral suppression. Table 3 illustrates the empirical clinical evidence taken from the 23 studies, with the detailed view presented in Online Appendix II. Prospective studies, such as randomised clinical trials and observational studies, tended to be restricted to only one or two areas of HIV and not to the complete continuum of care. Irrespective of the study considered, the implementation of payment models led either to a neutral or positive impact across the five areas of HIV considered (Table 3). As an example, a recent randomised clinical trial by El-Sadr et al. (2019) [19] assessed the impact of individual financial incentives in two areas of HIV care: continuity of healthcare (proportion of patients with CD4 + count in 4 of the last 5 quarters); and viral suppression (proportion of patients with a viral load below 400 copies per ml among patients with at least 2 counts in the last 5 quarters). A higher proportion of participants in the intervention group showed greater continuity of health care compared to the control group (7.5%, p = 0.007, t-test) and exhibited a trend towards improved HIV viral suppression (2.7%, p = 0.076, t-test).

### Economic Evidence

Regarding the magnitude of incentives, empirical evidence suggests that, regardless of the model’s recipient, the probability of influencing behaviours increases with the respective magnitude of the incentive (see Online Appendix II). In other words, the greater the incentive, the more likely to influence behaviours. Moreover, in the context of behavioural economics, more than the absolute value of the incentive, it is important to relate the magnitude of the incentive to the level of income of each participant (relative value of the incentive compared to the level of income of the participant). For example, studies carried out on the African continent presented lower incentives [between $ 1 and $ 40 when compared to high-income countries, such as the USA (average cost between $ 100 and $ 1173)], but their relative value compared to the respective average income per participant tends to be comparable or even higher.

Only one study analysed the durability of the results following the implementation of the payment model [19]. In this study, a randomised clinical trial [19], the findings from the payment model were sustained 9 months post its implementation.

### Critical Appraisal

The risk of bias associated with each study was assessed using the MMAT. Fifty-two percent (12/23) of papers included presented ‘high quality’, 43% (10/23) ‘intermediate quality’ and 4.3% (1/23) ‘low quality’ (Table 2). Randomised controlled trials and modelling studies (cost-effectiveness studies) scored higher compared to non-randomised trials.

## Discussion

Despite the design of the search terms, aimed at maximising the empirical evidence to include, the systematic literature review demonstrated the existence of limited empirical evidence around the implementation of payment models in the field of HIV and / or multimorbidities. The 23 articles included in the review showed important differences, concerning: (i) the country of origin; (ii) the type of study; (iii) recipient of the model; (iv) HIV care area considered; and (v) the type of intervention considered.

First, 57% (13/23) of the papers came from high-income countries, particularly the USA (11/13). The remaining 43% (10/23) studies come from seven countries on the African continent. The main difference between studies from low and high-income countries resided in the area of ​​HIV care that the model seeks to impact, and not the type of study or the intervention’s recipient. Studies from low-income countries tended to focus on HIV prevention, HIV testing and screening, linkage to HIV care and initiation of antiretroviral therapy, whilst high-income countries tended to target subsequent dimensions of the continuum of care, in particular health care retention, adherence to treatment and viral suppression. This evidence seems to be consistent with the key issues that the interventions tried to address. The high incidence and vertical transmission of HIV in African countries is the key issues that health systems currently face, whereas in high-income countries, health systems have more difficulties around the integration of care, retaining people with HIV in antiretroviral therapy and ultimately suppress viral load.

Second, there were no differences concerning the impact of different payment models depending on the type of study, with all studies showing either a neutral or positive impact.

Third, the interventions centred on two distinct levels of recipients, health organisations and health care users themselves. No study aimed to incentivise health professionals. Most evidence focused on health care users (83%), followed by health organisations (30%). The fact that most interventions targeted health users themselves, i.e. people at higher risk of HIV infection and/or people living with HIV infection, is aligned with behavioural economics theories [41] and reflects the individual importance of the user in the context of the entire care continuum. The inherent rationale is that health systems should devote financial resources to change the way users behave, seeking to enhance the cost-effectiveness of the care provided. Although this approach is consistent and independent of the health system considered, there is an important difference depending on the income level of the country of origin. Low-income countries aimed to promote cost-effectiveness by focusing on reducing infection rates and the initiation of antiretroviral therapy whilst high-income countries focused on promoting adherence to antiretroviral therapy and, ultimately, achieve viral suppression. The inherent rationale is to improve quality of life of people living with HIV, while avoiding costly episodes of hospitalisation in patients without viral suppression.

Fourth, five areas along the HIV care continuum were considered. As illustrated in Table 2, different papers focused on one or several areas, with 43% (10/23) of articles assessing the impact on viral suppression, followed by 30% (10/23) around HIV prevention and retention in healthcare and adherence to treatment and 22% (5/23) in the remaining areas (HIV testing and screening; link to HIV care and early antiretroviral therapy). It should be noted that the evidence surrounding viral suppression derived mainly from high-income countries.

Fifth, about the intervention, i.e. the payment model, this usually includes the use of financial incentives to encourage both health organisations and their users. These payments were mostly fixed, followed by conditional payments or based on lotteries. The use of conditional incentives is particularly relevant in: (i) care retention, where the user or organisation is only reimbursed according to consecutive indicators of access to care (e.g. consecutive CD4 counts); and (ii) viral suppression, where the user or organisation is only incentivised if viral suppression is achieved and/or sustained (e.g. CD4 counts below a given clinical objective). Contrary to some theory of behavioural economics, these incentives were always of a positive nature, that is, aimed at promoting behaviours, and not negative ones, which aim to inhibit behaviours (“carrot and not the stick”). A final point was the use of non-financial incentives (e.g. food items, smart phones) in studies developed in low-income countries.

Despite these methodological differences, the implementation of payment models presented a neutral or positive impact throughout the HIV care continuum. In fact, no study has shown a negative impact from applying the intervention with a payment model. This means that payment models improved the care provided under different dimensions of analysis, particularly in terms of access and quality of care. In particular, one high-quality randomised clinical trial conducted in the USA (HPTN 065 study) showed that the use of payment models led to a statistically significant improvement in the suppression of HIV viral loads [27]. These findings, consistent throughout the evidence analysed, seem to reflect the potential for pay-for-performance to improve HIV care. However, publication biases were not formally assessed.

Additionally, the financial impact of the intervention appears to be aligned with the financial sustainability of the health system itself. When considering the four studies that evaluated the cost-effectiveness of HIV payment models [38–41], these were found to have a high probability of being cost-effective taking into account the usual financial envelopes of the health systems (“willingness-to-pay thresholds”). This means that the interventions represent “value for money” and the increased costs associated with the payment model were more than offset by the avoided costs and / or by improving the quality of life of people living with HIV infection.

In terms of economic findings, the magnitude of the payment seems to be associated with the probability of success of the respective intervention. In the context of behavioural economics, more than the specific value of the incentive (absolute value), it is important to relate the magnitude of the incentive to the level of income of each participant (relative value of the incentive compared to the level of income of the participant). Finally, there was little evidence regarding the durability of the impact of the intervention. More research is needed to understand the long-term implications and sustainability of payment models in changing organisational and patients’ behaviours.

Lastly, payment models in the HIV domain are at an early stage of development. This situation is similar to the payment models used in the context of patients with multimorbidity [42]. However, due to the increasing chronicity associated with HIV, novel payment models should consider HIV in the context of other clinical conditions (e.g. diabetes, mental health). Also, general payment models hold the potential to indirectly affect HIV care. For instance, a payment model that incentivises healthy habits or diabetes care holds the potential to impact patients who also live with an HIV infection. For these reasons, further research on the real-world implementation and cost-effectiveness of new or novel payment models is strongly recommended.

### Review Limitations

The present review covers a period of 12 years (since 2008). The authors believe the exclusion of studies prior to 2008 constitutes a minor limitation as most relevant performance-based payment models, particularly in high-income countries, were constituted following the introduction of HIV antiretroviral drugs. The review includes only HIV specific performance-based payment models. Therefore, the potential impact of generic payment models across HIV patients is not included, which constitutes a review limitation. Given its broad inclusion criteria, all range and heterogeneity of studies conducted in different health care settings were included in the review. Despite this, a small number of studies (23) were included. The quality of these studies was assessed using the MMAT methodology, but no individual assessment of publication biases was conducted. Although a limitation, the authors believe that the review’s findings are likely to reflect the real-world implementation of HIV-based performance payment models. Lastly, given the non-comparability of the studies retrieved, we conducted a narrative review and not a meta-analysis.

## Conclusion

This review summarised the evidence around the use of performance-based payment models in the context of HIV and multimorbidity. Despite the very limited amount of evidence and its heterogeneity, the utilisation of payment models seemed to be associated with neutral or positive impact across different dimensions of HIV care and was likely to be cost-effective. However, the optimal magnitude of the incentives, as well as its long-term durability, remains unclear and therefore further research is recommended.

## Supplementary Information

Below is the link to the electronic supplementary material.

Supplementary file1 (DOCX 51 KB)

## Data Availability

Data available upon request.

## Abbreviations

ART: Antiretroviral treatment
MMAT: Mixed Method Appraisal Tool
USA: United States of America
UK: United Kingdom
