# Supplementary material for: The Utilisation of Payment Models Across the HIV Continuum of Care: Systematic Review of Evidence
Source: AIDS Behav. 2021 Jun 28;25(12):4193–208. doi: 10.1007/s10461-021-03329-2 (PMC8602234; doi:10.1007/s10461-021-03329-2)
Supplement: Supplementary file 1 — Supplementary file1 (DOCX 51 KB) [file 10461_2021_3329_MOESM1_ESM.docx]

**Supplementary material (Appendix I, II and III):**

**Appendix I:** Search strategy.

(HIV Infections[MeSH] OR HIV[MeSH] OR hiv[tiab] OR hiv-1*[tiab] OR hiv-2*[tiab] OR hiv1[tiab] OR hiv2[tiab] OR hiv infect*[tiab] OR "human immunodeficiency virus"[tiab] OR "human immunedeficiency virus"[tiab] OR "human immuno-deficiency virus"[tiab] OR "human immune-deficiency virus"[tiab] OR ((human immun*[tiab]) AND (deficiency virus[tiab])) OR AIDS[tiab] OR "acquired immunodeficiency syndrome"[tiab] OR "acquired immunedeficiency syndrome"[tiab] OR "acquired immuno-deficiency syndrome"[tiab] OR "acquired immune-deficiency syndrome"[tiab] OR ((acquired immun*[tiab]) AND (deficiency syndrome[tiab])) OR "sexually transmitted diseases, Viral"[MeSH:NoExp]) OR (Multimorb*[tiab] OR Multi-morb*[tiab]) AND (performance[tiab] OR results[tiab] OR output[tiab] OR delivery[tiab] OR conditional[tiab] OR contract*[tiab] OR economic*[tiab]) AND (financ*[tiab] OR subsid*[tiab] OR remunerat*[tiab] OR pay*[tiab] OR incentiv*[tiab] OR cash[tiab] OR commission*[tiab]​OR pric*[tiab]) AND ("2008"[PDAT] : "2020"[PDAT])

**Appendix II:** Clinical and cost evidence grouped per study design.

| Authors | Objectives  (primary and secondary)  Process vs. result indicators | Results (primary and secondary), (clinical and economic-financial) | Authors' conclusions (including sensitivity analyses) | Overall article quality |
| --- | --- | --- | --- | --- |
| **Randomised controlled trial** | | | | |
| Choko et al. (2019) | **Primary:** 1) proportion of male partners who have been tested for HIV and sought preventive care or treatment in the first 28 days after recruitment of the pregnant woman.  **Secondary:**  2) proportion of male partners who have been tested for HIV;  3) Average cost of the incentive model by randomization group (multiple interventions with different incentives) | **Primary:** 1) All interventions with financial incentives, except for the intervention with incentives based on a draw (p = 0.297), produced statistically significant differences (p <0.001) in the proportion of male partners who were referred for antiretroviral treatment or circumcision compared to the control group.  **Secondary:**  2) All interventions with incentives (financial and non-financial) produced, with the exception of the intervention with incentives based on a draw (p = 0.220), statistically significant differences (p <0.05) proportion of male partners who started treatment antiretroviral drugs or circumcised.  3) The average cost per partner tested for HIV ranged from $ 23.73 (HIV test + $ 3 incentive) to $ 41.24 (HIV test + telephone follow-up). The average cost of starting antiretroviral therapy or circumcision ranged from $ 94.32 (HIV test + $ 3 incentive) to $ 167.95 (HIV test + lottery-based incentive). These values ​​are higher compared to the control group, with an average cost per HIV tested partner of $ 9.85 and average cost of starting antiretroviral therapy or circumcision was $ 39.81. | The provision of rapid HIV tests to male caregivers of pregnant women has enhanced intervention programs in HIV prevention and treatment. In addition, the granting of financial incentives further enhances prevention, conducting screening tests, and treatment based on positive results, in particular adherence to antiretroviral therapy and male circumcision. | High |
| El-Sadr et al. (2019) | 1) Health care continuity (proportion of patients with CD4 + count in 4 of the last 5 quarters).  2) Suppression of HIV viral load 9 months post-recruitment (proportion of patients with a viral load below 400 copies per ml among patients with at least 2 counts in the last 5 quarters). | 1) A higher statistically significant proportion of participants in the intervention group showed greater continuity of health care compared to the control group (7.5%, p = 0.007).  2) There was a tendency for participants in the intervention group to have greater viral suppression of HIV compared to the control group (2.7%, p = 0.076). | The allocation of financial incentives has the potential to shape the behaviour of HIV patients 9 months after the end of the financial incentive allocation program (durability), in particular regarding continuity of care. | High |
| Thirumurthy et al. (2019) | Viral suppression (measured at baseline, and at 6, 12, 24 or 48 weeks).  **Primary outcome:** viral suppression (viral load <400 copies per mL) at 24 weeks;  **Secondary outcome:** viral suppression at 48 weeks. | - In the "intention-to-treat" analysis, 168 participants (84%) in the intervention group and 156 participants (82%) in the control group were virally suppressed at 24 weeks (odds ratio, OR, 1.14, 95% CI 0.68–1.93, p = 0.62).  - In the "per-protocol" analysis limited to participants with measurements of viral load at 24 weeks, there were also no differences between groups;  - The withdrawal of incentives at 24 weeks did not affect viral suppression, with 176 (88%) and 154 (81%) of participants in the intervention and control group, respectively, being viral suppressed at 48 weeks. | Financial incentives had no effect on viral suppression in HIV-positive adults. The results suggest that achieving viral suppression requires more intensive interventions, in addition to financial incentives. | Intermediate |
| Kadota et al. (2018) | Adherence to care (adherence to ART), assessed by achieving the rate of possession of medicines ≥ 95% at 6 and 12 months.  Link and retention in HIV care, assessed through the number of patients lost to follow-up at 12 months. | Short-term transfers:  1) improved the rate of possession of medicines ≥ 95% at 6 and 12 months;  2) reduced patients lost to follow-up in most subgroups.  Subgroup analyses  They were conducted according to gender, age, wealth, and time between the diagnosis of HIV and the ART induction. They showed that the wealth and the time between the diagnosis and the start of ART, are potential effect-modifying measures, with greater effects for the possession of medicines ≥ 95% at 6 months in the poorest patients, compared with the richest, and in recently diagnosed individuals (<90 days since diagnosis), compared to those ≥90 days old, patterns that were maintained at 12 months. | The authors concluded that transfers of food and money may have stronger beneficial effects on adherence to ART in the poorest patients and suggested that it may be important to target interventions for patients most recently diagnosed with HIV. | High |
| Maughan-Brown et al. (2018) | 1) linkage to care (defined as visiting the health institution within 3 months after being included in the study);  2) initiation of ART (initiation of treatment within 3 months) | 67% of participants remain linked to care and 42% started ART in the 3 months following referral.  No significant differences were found between groups, in terms of the linkage to care or the initiation of ART.  Ordinary regression analyses showed that encouraged individuals were connected to care and started treatment in less days, but none of these results were statistically significant. | Conditional cash incentives do not appear to improve the proportion of participants linked to care or who start ART within 3 months, even though the individuals encouraged appear to have sought care and treatment more quickly (non-significant result). | Intermediate |
| Mills et al. (2018) | **Clinical**:  Primary outcomes: changes in CD4 + cell count, adherence to ART, and sexual behaviours.  Secondary outcomes: changes in household food security, mental health.  **Economic**:  Health expenses. | **Clinical**: There were no significant effects of the intervention on CD4 + T cell counts between groups, on food safety, medication adherence, or sexual behaviours;  - In secondary analyses, an effect of mental planning was detected in changing the CD4 + cell count between groups;  **Economic:** The average change in health spending was $ 2.65 (95% CI: 9.30 to 15.69) dollars spent in the previous 3 weeks with a 0.66 chance of a positive effect from the intervention. | No effects of unconditional monetary incentives were found in several health outcomes analysed in HIV-infected patients in rural Uganda.  The authors point out, however, that the results may be different in other contexts, mainly because the individuals in this study already had medical monitoring and access to antiretrovirals. | Intermediate |
| Montoy et al. (2018) | **Primary outcome:**  proportion of patients who agreed to take an HIV test | - 55.4% of all patients accepted an HIV test; patients who were offered no monetary incentive accepted 51.6% of the tests; those offered incentives of $ 1, $ 5, and $ 10, 52.6%, 62.1%, and 66.6% of patients agreed to take the test, respectively.  - the $ 1 incentive did not increase test acceptance; the $ 5 and $ 10 incentives increased acceptance rates by 10.5 and 15%, respectively;  - compared to the "opt-in" test option, the "active-choice" test option increased test acceptance by 11.5% (95% CI 9.0 to 14.0), and the "opt-out test option" increased acceptance by 23.9% (95% CI 21.4 to 26.4). | The authors found evidence that two economic-behavioural interventions [monetary incentives and standard test options (defaults)] can be effective in increasing the rate of HIV testing. Among all the tested assignments, the "opt-out" option had the greatest effect, followed by the $ 10 incentive. | High |
| Chamie et al. (2018) | **Clinical:**  Conducting an HIV test in a community health campaign (primary outcome) | - of the 2,532 participants, 1,924 (76%) underwent HIV testing; 7.6% of those tested were HIV positive.  - there were no significant differences in test acceptance in the two groups of incentives based on raffles, or in the two groups with incentives framed in the loss vs. two groups with incentives framed in the gain.  - among the types of incentives, testing did not differ significantly between the high-cost group (76%) vs. the low-cost group (75%); among low-cost groups, acceptance of tests was significantly higher in the group with incentives based on raffles (80%) vs. group with incentives framed in the gain (72%). | The use of incentives based on draws, as well as providing incentives framed in loss, have not resulted in significant increases in HIV testing compared to standard incentives framing gains. However, by offering low-cost incentives to promote HIV testing, providing incentives based on draws may be a better strategy than incentives based on earnings. | High |
| Alsan et al. (2017) | **Clinical:**  Suppression of viral load (pVL≤200 copies / mL) at the end of the incentive period and on an unscheduled post-incentive visit in the following three months. | The probability of viral suppression was higher in the incentive choice group than in the passive control group at the post-incentive visit (adjusted odds ratio of 3.93; 95% CI 1.19 - 13.04; p = 0.025). The differences in relation to the passive control group at the end of the incentive period and in relation to the incentive group to visit professionals in the two moments of assessment were not statistically significant.  After the incentives for adhering to ART and participating in visits to professionals were removed, participants who were offered a commitment contract for adhering to ART were more likely to achieve viral suppression compared to individuals who were allocated to routine care, despite the difference being only statistically significant in the last comparison. | Commitment contracts can improve adherence to ART and viral suppression. | Intermediate |
| El-Sadr et al. (2017) | 1) Adherence to health care: proportion of HIV patients (new HIV patients or not connected to health care in the last 12 months) with access to health care in the last 3 months (CD4 test)  2) Suppression of HIV viral load: proportion of HIV patients (established HIV patients) with suppressed viral load (CD4 <400 copies / mL) each quarter.  3) Health care continuity: proportion of HIV patients (established HIV patients) with CD4 counts during at least 4 of the last 5 quarters. | 1) The allocation of financial incentives did not significantly increase adherence to healthcare compared to the control group (odds ratio 1.10, 95% CI: 0.73-11.67, p = 0.65)  2) At baseline, the proportion of patients with suppressed viral load was 62%. The proportion of patients with suppressed viral load increased in both groups (intervention and control), with the difference between groups being statistically significant, that is, the allocation of financial incentives increased the proportion of patients with suppressed viral load by 3.8% (05 % CI: 0.7% -6.8%, p = 0.01).  3) Continuity of health care: The allocation of financial incentives increased the proportion of patients with continuity of health care (increase of 8.7%, 95% CI: 4.2% -13.2%, p <0.001)  Subgroup analyses for objective 2:  a) The suppression of viral load was statistically significant in: i) health care providers in Washington (6.6%, 95% CI: 1.9% -11.3%, p = 0.006); ii) hospital health care providers (4.9%, 95% CI: 1.4% -8.5%, p = 0.007); iii) providers with lower levels of viral suppression when recruiting (5.6%, 95% CI: 0.0% -11.3%, p = 0.05); and iv) providers with higher levels of viral suppression when recruiting (increase of 3.6%, 95% CI: 0.3% -7.0%, p = 0.03).  b) There are no statistically significant differences in New York providers or depending on the size of the provider.  c) Among patients with no viral load suppressed at recruitment, the allocation of financial incentives produced a difference between groups (4.9%, 95% CI: 1.4% -8.5%, p = 0.007). | The study (HPTN 065) was, at the time of its publication, the largest study to assess the effectiveness of financial incentives on indicators associated with the provision of health care in ​​HIV. Although apparently modest, a 4% increase in the proportion of patients with suppressed viral load has considerable clinical and prevention implications, particularly in geographical areas and / or patients with a lower proportion of viral load suppression.  The authors recommend further studies to determine the feasibility of carrying out incentive programs on a larger scale. | High |
| Stitzer et al. (2017) | 1) Presence in the patient's browsing sessions  2) Viral suppression at the end of the intervention (compared to individuals with low, medium and high presence) | 1) most participants in the group with only patient navigation were present in 6 or more sessions, but only 28% were present in 10 or more, and 16% were present in all 11 sessions. In contrast, 90% of participants in the patient navigation + financial incentives group were present in 6 or + sessions, 69% were present in 10 or more, and 57% were present in all 11 sessions.  2) the percentage of viral suppression at 6 months was 15, 38 and 54% among those who were present at 0–5, 6–9 and 10–11 visits, respectively. The average number of assisted sessions differed significantly between the 2 groups (the average number of assisted sessions was 7 for the group with only patient navigation, while in the navigation + financial incentives group it was 11). A significant association was found between viral suppression and session attendance rates. | Financial incentives for attendance at the patient's browsing sessions significantly improved contact between individuals with HIV and a history of substance use and their healthcare contacts who provided an intervention designed to encourage re-engagement with HIV health care and with support services in the use of substances. | Intermediate |
| Metsch et al. (2016) | **Primary**: viral suppression of HIV vs non-viral suppression of HIV or death at 12 months.  **Secondary**: (i) HIV-related (e.g., viral suppression of HIV within 6 months, outpatient care with a specialist, medication adherence); (ii) related to substance use (were assessed at 6 and 12 months) | **Primary objective:** no differences in HIV viral suppression rates versus non-suppression or death between the three groups at 12 months.  **Secondary objectives:** (i) HIV-related - none of the results at 12 months were statistically different by treatment group; (ii) related to substance use - there were no significant differences between groups in the results of urine, nor in self-reported days of substance use at 6 or 12 months. | The two interventions did not result in higher rates of suppression in relation to non-viral suppression or death at 12 months of follow-up, that is, 6 months after the completion of the interventions. In all study groups, just over a third of participants achieved viral suppression at 12 months. | High |
| de Walque et al. (2015) | 1) Number of users tested for HIV  2) Number of couples tested during a month after contacting the provider | 1) Trend of increase of 6.1% (p = 0.126) in the proportion of users tested for HIV in the intervention group (incentive for the health organisation of $ 0.92 per user tested).  2) Increase of 14.5% (p <0.05) in the proportion of couples tested for HIV in the intervention group (incentive for the health organisation of $ 4.59 per couple tested) | The use of a pay-for-performance payment models aimed at health care organisations has increased the likelihood of patients, in particular couples, being tested for HIV. In addition, the magnitude of the incentives appears to be related to their respective impact. | Intermediate |
| de Walque et al. (2012) | Clinical: Prevalence of four sexually transmitted diseases and HIV in screening tests every 4 months (up to a total of 12 months)  Economic: Average cost of the incentive model in both groups. | **Clinical**: The use of incentives in the high incentive group produced a statistically significant difference in the prevalence of sexually transmitted diseases 12 months post-recruitment compared to the control group. These results were not replicated in the low incentive group or the high incentive group during the first 8 months post-recruitment (testing at 4 and 8 months).  **Economic**: The results of the average cost are not explicitly presented, but it is estimated at around $ 30 / $ 60 for the group with low / high level of incentive, respectively. | The use of conditional financial incentives has the potential to reduce the prevalence of sexually transmitted diseases (including HIV infection). However, only the group with the highest level of incentives showed a statistically significant difference at 12 months post-recruitment. | Intermediate |
| Barnett et al. (2009) | Clinical:  1) Adherence to medication  Economic:  1) Costs of screening, medication coaching, and vouchers - the cost of replicating the intervention in clinical practice (from the payer's perspective) was estimated.  2) Cost of health services within the system - data were collected on all costs of health care and substance abuse treatment  3) Cost of health services outside the system (e.g., cost of visits to the emergency department, medical visits, consultations at a mental health clinic) | **Clinical**:1) Participants randomised to incentives in the form of vouchers were significantly more adherent to medication during the 12 weeks of the intervention period. During the 4-week post-intervention period, adherence assessed by electronic monitoring dropped to 66% in the incentive group, which was not significantly greater than the 53% adherence seen in the comparison group (p = 0.07).  **Economic**: 1) Screening during the initial 4-week observation period cost $ 42.53 per patient, including assessing the participant's eligibility, identifying the medication to monitor, and monitoring.  2) During the 12-week intervention period, the direct incremental cost of the intervention, including vouchers, was $ 942 ($ 66 for the initial screening; $ 378 value of  vouchers, $ 416 for administrative voucher costs and $ 81 for membership assessments and follow-up reminders). The voucher group incurred $ 2,572 in costs with antiretroviral medication, significantly higher than the $ 1,973 incurred in the comparison group (p <0.01); | Voucher incentives can improve adherence to antiretroviral therapy in HIV-positive patients enrolled in methadone maintenance programs. Despite the long-term improvements in the health of the intervention, these benefits cannot be sufficient to justify its cost. | Intermediate |
| **Non-randomised prospective study** | | | | |
| Belenky et al. (2018) | **Clinical**: 1) adherence to antiretroviral therapy; 2) viral suppression.  **Economic**: 3) out-of-pocket cost borne by patients on prescription drugs; and 4) use of HIV support programs; | **Clinical**: There were no statistically significant differences between groups in terms of adherence to antiretroviral therapy (difference of 4%, p = 0.44) nor in suppression of viral load (difference of 0.1%, p = 0.987).  **Economic**: there were statistically significant differences between groups in terms of out-of-pocket spending (20% difference, p <0.001) and in access to financial support programs for HIV patients (10% difference, p = 0.007). | The new Medicare Part D payment model led to an increase in out-of-pocket spending but this was not associated with statistically significant differences in adherence to antiretroviral therapy nor viral suppression. | High |
| Brantley et al. (2018) | **Clinical**: 1) Adherence to health care; 2) Suppressed viral load.  **Economic**: 3) Average incentive level (in USD $) per patient included in the payment model with individual incentives. | **Clinical**: 1) 97.6% / 95.1% of patients had at least a CD4 count in the 12/24 months post-recruitment; 2) suppression of viral load increased statistically significantly (82.7% vs 57.8%, p <0.001). All analysis subgroups, except for patients with suppressed viral load at the time of recruitment for the study, showed a statistically significant difference in suppression of viral load.  **Economic**: 3) the average incentive per patient was $ 160.81. | The allocation of individual incentives contributes to the improvement of clinical indicators, both in terms of process and outcomes, at an average cost per patient financially affordable. | Intermediate |
| Rajkotia et al. (2017) | 1) indicators related to maternal and child health.  2) indicators related to the prevention of mother-to-child transmission.  3) paediatric HIV indicators | **1, 2 and 3)** Performance-based payment models presented positive impacts on maternal and child health, prevention of mother-to-child transmission, and paediatric indicators in HIV programs.  Subgroup analyses: The comparison between institutions in the North and institutions in the South, found that the context in which it is applied is an important determinant of its success; differences were found between the two regions, as well as differences in the magnitude of the responses. | The performance-based payment program in Mozambique produced large and sustained increases in the provision of prevention services for mother-to-child transmission, paediatric HIV services, and maternal and child health services. The authors concluded that performance-based payment models are an effective strategy to reduce the HIV epidemic and improve maternal and child health care. | High |
| Foster et al. (2014) | 1) HIV viral load at different time points: 2 and 4 weeks, 3, 6, 12- and 24-month post-intervention.  2) Average incentive per participant with HIV. | 1) 12 months post-recruitment, 9 (out of 11) participants showed an improvement in the CD4 count compared to the initial count. 24 months post-recruitment (12 months after the end of the incentive model), 5 (out of 10) participants showed an improvement in the CD4 count compared to the initial count.  2) Average incentive per patient of £ 121 (minimum £ 0, maximum £ 200). The model for the 11 participants led to a total cost of the incentive model of £ 1,350. | The use of financial incentives in combination with motivational interviewing techniques has the potential to improve clinical indicators without excessive cost. The authors recommend further research to assess the impact of this pilot. | Low |
| **Modelling studies** | | | | |
| Wagner et al. (2020) | **Clinical**:  *Primary*: Number of new HIV diagnoses;  *Secondary*: infections prevented  **Economic**: cost of programs, from the payer's perspective (hospital) | **Clinical**: *Primary*: Moving from the "opt-in" option to the "opt-out" option resulted in 39 additional diagnoses (56% increase) after 1 year. Moving from no incentive to a $ 1, $ 5, or $ 10 incentive increases 14, 13 and 28 new diagnoses, respectively (20, 19, and 41% increase), respectively.  *Secondary*: No differences were found in terms of avoided infections.  **Economic:** Moving from the "opt-in" option to the "opt-out" option costs $ 3807 per new diagnosis. Moving from no incentive to a $ 1, $ 5, or $ 10 incentive has a cost of $ 11,050, $ 17,984, and $ 15,298 per new diagnosis, respectively. | From the hospital's perspective, the most effective policies for identifying infected patients involved changing the way screening offerings were structured, rather than offering financial incentives to individuals. | Intermediate |
| Adamson et al. (2019) | **Primary**: Cost per QALY from the perspective of the payer and society  **Secondary**: Cost per QALY from the perspective of society | **Primary**: additional cost per QALY of $ 49,877.  **Secondary**: intervention is dominant (gain of 0.06 QALYs per patient at a cost of less than $ 4,210) | The allocation of marginal individual incentives allowed an improvement in the viral load of the disease (CD4), allowing an extension of the life of the HIV patient. Deterministic and probabilistic sensitivity analyses estimated that the intervention has a 73% probability of being cost-effective. | High |
| Stevens et al. (2018) | **Clinical**:  1) new HIV infections  2) prevented deaths  **Economic**:  1) cost per prevented infection  2) QALY | **Clinical**:  1) the Link4Health strategy would reduce new infections in 20 years, by 11,059 infections, a 7% reduction in relation to the 169,019 projected cases  2) the Link4Health strategy would prevent 5,313 deaths, an 11% reduction from the 49,582 projected deaths.  **Economic**:  1) the Link4Health program resulted in an incremental cost per prevented infection of $ 13,310  2) the Link4Health program resulted in an incremental cost per QALY earned of $ 3,560 / QALY from the health sector perspective | The Link4Health strategy is likely to be a cost-effective strategy for responding to the HIV epidemic in Swaziland. | High |
| Heymer et al. (2012) | **Clinical**:  Number of new transmissions  **Economic**:  1) Difference in QALYs  2) costs of co-payments for ART vs. treatment costs | **Clinical**:  1) It has been estimated that one HIV infection is avoided per year for every 31.4 people (median, 24.0-42.7 interquartile range) receiving treatment.  **Economic**:  1) a prevented infection leads to approximately 7.5 QALY gains. Therefore, the approximate cost per QALY is US $ 58,613 (= US $ 439 600 / 7.5), which is below the limit of the amount referred to as willingness to pay.  2) When considering the incremental change in costs and the results of a change to the current program, it was found that this would cost the health sector US $ 17,860 per prevented infection (median, US $ 13 651–24 287 IQR) if ART is provided as a combination of three doses and three drugs, with no requirements for co-payments paid by the user. | It would be a cost-effective option for the Australian government to cover the financial costs of co-payments for antiretroviral drugs in HIV in the epidemiological context of South Australia. | High |
